# Novel Visible Light-Driven Ho_2_InSbO_7_/Ag_3_PO_4_ Photocatalyst for Efficient Oxytetracycline Contaminant Degradation

**DOI:** 10.3390/molecules30153289

**Published:** 2025-08-06

**Authors:** Jingfei Luan, Tiannan Zhao

**Affiliations:** 1 School of Physics, Changchun Normal University, Changchun 130032, China; 15734278033@139.com; 2 State Key Laboratory of Pollution Control and Resource Reuse, School of the Environment, Nanjing University, Nanjing 210093, China

**Keywords:** Ho_2_InSbO_7_/Ag_3_PO_4_ heterojunction, direct Z-scheme, oxytetracycline, photocatalytic efficiency, degradation mechanism, degradation pathway

## Abstract

In this study, a Z-scheme Ho_2_InSbO_7_/Ag_3_PO_4_ (HAO) heterojunction photocatalyst was successfully fabricated for the first time by ultrasound-assisted solvothermal method. The structural features, compositional components and morphological characteristics of the synthesized materials were thoroughly characterized by a series of techniques, including X-ray diffraction, Fourier transform infrared spectroscopy, Raman spectrum, X-ray photoelectron spectroscopy, transmission electron microscopy, scanning electron microscopy and energy-dispersive X-ray spectroscopy. A comprehensive array of analytical techniques, including ultraviolet-visible diffuse reflectance absorption spectra, photoluminescence spectroscopy, time-resolved photoluminescence spectroscopy, photocurrent testing, electrochemical impedance spectroscopy, electron paramagnetic resonance, and ultraviolet photoelectron spectroscopy, was employed to systematically investigate the optical, chemical, and photoelectronic properties of the materials. Using oxytetracycline (OTC), a representative tetracycline antibiotic, as the target substrate, the photocatalytic activity of the HAO composite was assessed under visible light irradiation. Comparative analyses demonstrated that the photocatalytic degradation capability of the HAO composite surpassed those of its individual components. Notably, during the degradation process, the application of the HAO composite resulted in an impressive removal efficiency of 99.89% for OTC within a span of 95 min, along with a total organic carbon mineralization rate of 98.35%. This outstanding photocatalytic performance could be ascribed to the efficient Z-scheme electron-hole separation system occurring between Ho_2_InSbO_7_ and Ag_3_PO_4_. Moreover, the adaptability and stability of the HAO heterojunction were thoroughly validated. Through experiments involving the capture of reactive species and electron paramagnetic resonance analysis, the active species generated by HAO were identified as hydroxyl radicals (•OH), superoxide anions (•O_2_^−^), and holes (h^+^). This identification provides valuable insights into the mechanisms and pathways associated with the photodegradation of OTC. In conclusion, this research not only elucidates the potential of HAO as an efficient Z-scheme heterojunction photocatalyst but also marks a significant contribution to the advancement of sustainable remediation strategies for OTC contamination.

## 1. Introduction

To address the escalating demands in modern medicine, agriculture, and animal husbandry, significant quantities of antibiotic products are being used [1,2]. Oxytetracycline (OTC), a representative antibiotic from the tetracycline class, has been extensively utilized due to its broad-spectrum antibacterial activity and cost-effectiveness in treating diseases in both humans and animals, as well as in agricultural and aquaculture settings [3,4,5]. However, due to the low absorption efficiency of OTC in humans and animals, a substantial portion of unmetabolized OTC is excreted via feces and urine, subsequently entering aquatic ecosystems [6]. The persistence of OTC residues may disrupt ecological balance by promoting the dissemination of antibiotic-resistant genes, thereby presenting significant health risks [7,8,9]. Therefore, it is imperative to address the threats posed by OTC residues to human health and ecological systems.

Previous studies have extensively explored various remediation strategies for OTC contamination in environmental systems. However, conventional wastewater treatment technologies, including physical adsorption, chemical coagulation, and biofilm-mediated degradation, often demonstrate limited efficacy in the complete removal of OTC due to its chemical stability and resistance to biological degradation [10,11,12,13]. Hence, developing efficient, stable, and eco-friendly technologies for OTC removal remains a critical endeavor.

In contrast to traditional water pollution remediation technologies, photocatalytic advanced oxidation processes (PAOPs) offer distinct advantages, such as environmental benignity, cost-effectiveness, and superior degradation efficiency for persistent organic pollutants [14,15,16]. Photocatalysts play a pivotal role in these processes. Early investigations primarily focused on conventional single metal oxide photocatalysts, notably titanium dioxide (TiO_2_) and zinc oxide (ZnO). These semiconductor photocatalysts, characterized by wide band gaps, could only be photoexcited by ultraviolet (UV) light. UV light makes up around 4% of the total solar energy. As a result, there is considerable energy loss from the visible light spectrum, which approximately accounts for 43% of solar energy [17,18,19]. Recently, A_2_B_2_O_7_-type photocatalysts have drawn substantial attention owing to their stable pyrochlore structures and higher visible light utilization efficiency [20,21,22,23,24]. For example, Hao et al. reported that the photocatalytic removal efficiency (PRE) of acetochlor was 82.19% when using GdYBiNbO_7_ powder under visible light irradiation (VLI) [25]. Similarly, the photocatalysts synthesized by Li et al. exhibited a PRE of 74.60% for Rhodamine B within 240 min [26].

Bi_2_InNbO_7_ has shown tunable potential for structural modification, enabling enhanced photocatalytic performance through strategic ion substitution [27]. Moreover, prior studies have indicated that Ho-doped TiO_2_ significantly enhances photocatalytic activity for methyl orange [28], while the photocatalytic degradation rate of methylene blue was accelerated by Sb-doped TiO_2_ [29]. Consequently, a novel photocatalyst, Ho_2_InSbO_7_, was developed by incorporating Ho^3+^ and Sb^5+^ into Bi_2_InNbO_7_, which is anticipated to exhibit enhanced photocatalytic performance.

Nonetheless, single-component photocatalytic materials face significant limitations in practical applications, primarily attributed to the high recombination rates of photogenerated carriers (PGCs) [30,31]. To overcome the limitation, binary heterojunction photocatalytic materials have been proposed, featuring an internal electric field that effectively accelerates the spatial separation of photogenerated electron-hole pairs [32,33,34,35,36,37]. Compared to single-component photocatalytic materials, these heterojunctions demonstrate significantly enhanced photocatalytic performance in degradation experiments. For instance, ZnO/CuO heterojunctions synthesized by Pal et al. achieved a degradation rate of 98% for methyl orange within 280 min, outperforming pure ZnO [38]. Similarly, Qian et al. synthesized a Bi_4_O_5_Br_2_/Bi_2_WO_6_ heterojunction, achieving a degradation rate of 91.0% for ciprofloxacin under VLI, surpassing pure Bi_4_O_5_Br_2_ (74.1%) and Bi_2_WO_6_ (51.3%), with significantly improved reaction rates [39]. The optimized NiO/BiOI heterojunction catalyst developed by Hu et al. achieved nearly complete degradation of Rhodamine B within a 60 min reaction period [40].

Ag_3_PO_4_, which crystallizes in a body-centered cubic structure (P4-3n space group), is a promising photocatalyst [41,42,43]. Due to its suitable band energy position, it is widely utilized in the construction of heterojunction photocatalytic systems. For instance, Zhu et al. successfully synthesized a ternary heterojunction ZnO/GO/Ag_3_PO_4_, which exhibited a remarkable degradation efficiency of 96.32% for tetracycline (TC) when subjected to VLI over a period of 75 min [44]. In a comparable study, Zhang et al. developed a heterojunction Bi_2_MoO_6_/WO_3_/Ag_3_PO_4_ achieving an impressive degradation rate of 97.31% for Reactive Black 19 (RB-19) within a treatment duration of 2 h [45].

Given the aforementioned analysis and guided by an appropriate band structure, a novel visible light-driven direct Z-scheme Ho_2_InSbO_7_/Ag_3_PO_4_ (HAO) heterojunction photocatalyst was successfully synthesized for the first time via an ultrasound-assisted solvothermal method. A comprehensive set of analytical techniques, including X-ray diffraction (XRD), Fourier transform infrared spectroscopy (FTIR), Raman spectroscopy, X-ray photoelectron spectroscopy (XPS), transmission electron microscopy (TEM), scanning electron microscopy (SEM), energy dispersive spectroscopy (EDS), ultraviolet-visible diffuse reflectance absorption spectra (UV-Vis DRS), photoluminescence spectroscopy (PL), time-resolved photoluminescence spectroscopy (TRPL), photocurrent testing (PC), electrochemical impedance spectroscopy (EIS), electron paramagnetic resonance (EPR), and ultraviolet photoelectron spectroscopy (UPS), were systematically employed to investigate the structure, composition, morphology, and chemical and photoelectronic properties of the synthesized material. The operational feasibility of the direct Z-scheme mechanism was clearly confirmed. Experimental results demonstrated that the HAO heterojunction exhibited outstanding photocatalytic performance, achieving a removal efficiency of 99.89% for OTC within 95 min under VLI. The structural integrity was confirmed through cyclic stability tests. Additionally, this study elucidated the active species involved in the OTC degradation process, specifically hydroxyl radicals (•OH), superoxide anions (•O_2_^−^), and photogenerated holes (h^+^), and proposed a detailed photocatalytic degradation mechanism and corresponding degradation pathways. These findings highlight the superior efficacy and innovative potential of the HAO heterojunction catalyst in OTC degradation, laying the foundation for its application as an advanced photocatalyst in energy conversion and environmental remediation fields.

## 2. Results and Discussion

### 2.1. Characterization of Photocatalysts

**Figure 1:**
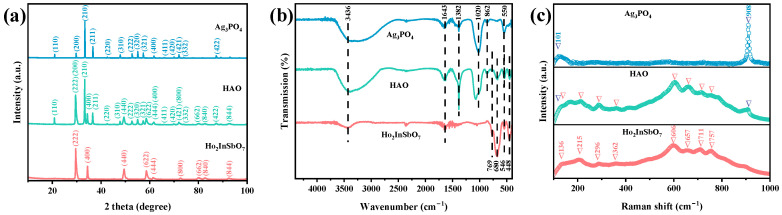
(**a**) XRD, (**b**) FTIR, and (**c**) Raman plots of Ho_2_InSbO_7_, Ag_3_PO_4_, and HAO.

Figure 1a illustrates the XRD profiles of the photocatalysts Ho_2_InSbO_7_, Ag_3_PO_4_, and HAO heterojunction. The diffraction data for Ho_2_InSbO_7_ and Ag_3_PO_4_ were obtained through independent XRD experiments, revealing sharp diffraction peaks that closely match their respective standard spectra. This observation indicates that both samples possess pure phases and identifiable crystalline structures. Additionally, the nanocomposite HAO displays distinct diffraction peaks corresponding to all characteristic reflections and crystal plane indices (hkl) of Ho_2_InSbO_7_ and Ag_3_PO_4_, with no extra peaks suggesting impurities. These results robustly confirm the successful construction of the HAO heterostructure and its high purity characteristics. To further validate the crystal structures, Rietveld refinement analyses were conducted on the XRD data of Ho_2_InSbO_7_ and Ag_3_PO_4_ using the Materials Studio software (Version 2.2). The refinement results are presented in Figures S1a and S2a. The experimental data and theoretical models exhibited a strong degree of consistency, as reflected by the Rp values of 7.08% for Ho2InSbO7 and 11.95% for Ag_3_PO_4_, respectively. This confirms the burnt greenstone-type crystal structure of Ho_2_InSbO_7_ and the body-centered cubic structure of Ag_3_PO_4_. Furthermore, the Ho_2_InSbO_7_ photocatalyst belongs to the Fd3m space group, while the Ag_3_PO_4_ photocatalyst falls under the P4-3n space group. The unit cell parameters for Ho_2_InSbO_7_ were determined to be 10.449 Å, while those for Ag_3_PO_4_ were a = b = c = 6.040 Å. The atomic structure models constructed based on the aforementioned space group assignments, crystal system types, lattice constants, and the atomic coordinates and structural parameters listed in Tables S1 and S2 are shown in Figures S1b and S2b. These extensive analyses furnish robust proof of the synthesized materials’ structural integrity and underscore their potential as highly efficient photocatalysts across various applications.

The pronounced distortion exhibited by the M(1)O_6_ octahedra (where M(1) = In^3+^ and Sb^5+^) within the crystal structure of Ho_2_InSbO_7_ reflects the characteristic distortion features of the lattice structure. Specifically, the compound Ho_2_InSbO_7_ contains two distinct types of Ho-O bonds: the longer bond, designated as Ho-O(1), has a length of 2.599 Å, while the shorter bond, referred to as Ho-O(2), measures 2.262 Å. Research indicates that discrepancies in bond lengths such as these can lead to a decreased recombination rate of PGCs, thereby enhancing the photocatalytic activity of the material [46]. It is particularly noteworthy that the M-O-M bond angle configuration directly influences the migration pathways of photoinduced charge carriers and their ability to reach active surface sites, ultimately determining the material’s photocatalytic performance. Existing studies have shown that photocatalytic activity is optimized when the bond angle approaches 180° [47]. In the case of Ho_2_InSbO_7_, the measured M-O-M angle is 134.020°, and this relatively large angle further augments the photocatalytic efficacy of Ho_2_InSbO_7_. This critical structural information is essential for understanding the catalytic mechanisms at play within this compound.

Figure 1b presents the analysis results of the chemical bond types and structural characteristics of the HAO heterojunction and the single-component photocatalysts Ho_2_InSbO_7_ and Ag_3_PO_4_ using FTIR. The FTIR absorption profile of HAO heterojunction retains the characteristic absorption peaks of both Ho_2_InSbO_7_ and Ag_3_PO_4_. Specifically, the peak at 448 cm^−1^ corresponds to the bending vibrations of the Sb-O bond [48], while that at 546 cm^−1^ reflects similar vibrations in the Ho–O bond [49]. The absorption peak observed at 769 cm^−1^ was linked to the stretching vibrations of the In–O bond [50], and the peak at 680 cm^−1^ was related to the vibrational bending of Sb-O-Sb bonds. Furthermore [51], the band at 550 cm^−1^ represents the asymmetric and symmetric stretching vibrations of the O=P–O bond [52]. The two bands observed at approximately 862 cm^−1^ and 1020 cm^−1^ could be attributed to the symmetric and asymmetric stretching vibrations of the O–P–O bond [52], while the peak at 1382 cm^−1^ corresponds to the stretching vibrations of the P=O double bond within the phosphate group [52]. It was noteworthy that all samples exhibit a broad absorption band at 3436 cm^−1^ in their FTIR spectra, which could be attributed to the vibrational response of the O–H bonds in water molecules [53]. The characteristic absorption peak at 1643 cm^−1^ corresponds to the bending vibration mode of the H-O-H bond [53]. A comprehensive analysis indicates that the systematic distribution of these FTIR characteristic peaks not only confirms the stable existence of the HAO heterojunction structure but also provides empirical evidence for a more in-depth understanding of the interfacial interaction mechanisms within this composite material and the optimization of its photocatalytic performance.

Figure 1c displays the Raman spectrum of the synthesized materials, highlighting several significant features indicative of the structural characteristics. Notably, peaks observed at 136 cm^−1^ and 215 cm^−1^ correspond to the vibrational bending modes connected with the Sb–O–Sb bond [54]. A peak at 362 cm^−1^ was linked to the tensile vibrations of Ho–O bonds [55], while the In–O–In stretching mode was observed at 296 cm^−1^ [56]. The Raman band at 606 cm^−1^ was associated with the stretching vibrations of the octahedral units (u(InO_6_)) [56]. Moreover, the In-O vibrational mode was observed at 657 cm^−1^ [57] and the peaks at 711 cm^−1^ and 757 cm^−1^ are ascribed to the T2 symmetry of the Sb–O–Sb band and the tensile vibrations of Sb–O bonds [58,59], respectively. The sharp and intense peak observed at 908 cm^−1^ corresponds to the symmetrical stretching of the terminal oxygen atoms in the [PO_4_] clusters [60]. Bands detected at 101 cm^−1^ were linked to the pure rotation or translation of the [PO_4_] units [60]. In summary, the Raman spectrum of HAO revealed a series of prominent peaks at 101 cm^−1^, 136 cm^−1^, 215 cm^−1^, 296 cm^−1^, 362 cm^−1^, 606 cm^−1^, 657 cm^−1^, 711 cm^−1^, 757 cm^−1^, 908 cm^−1^. In conclusion, the combination of XRD data with FTIR and Raman spectroscopic results offers robust proof for the successful fabrication of the HAO heterojunction.

**Figure 2:**
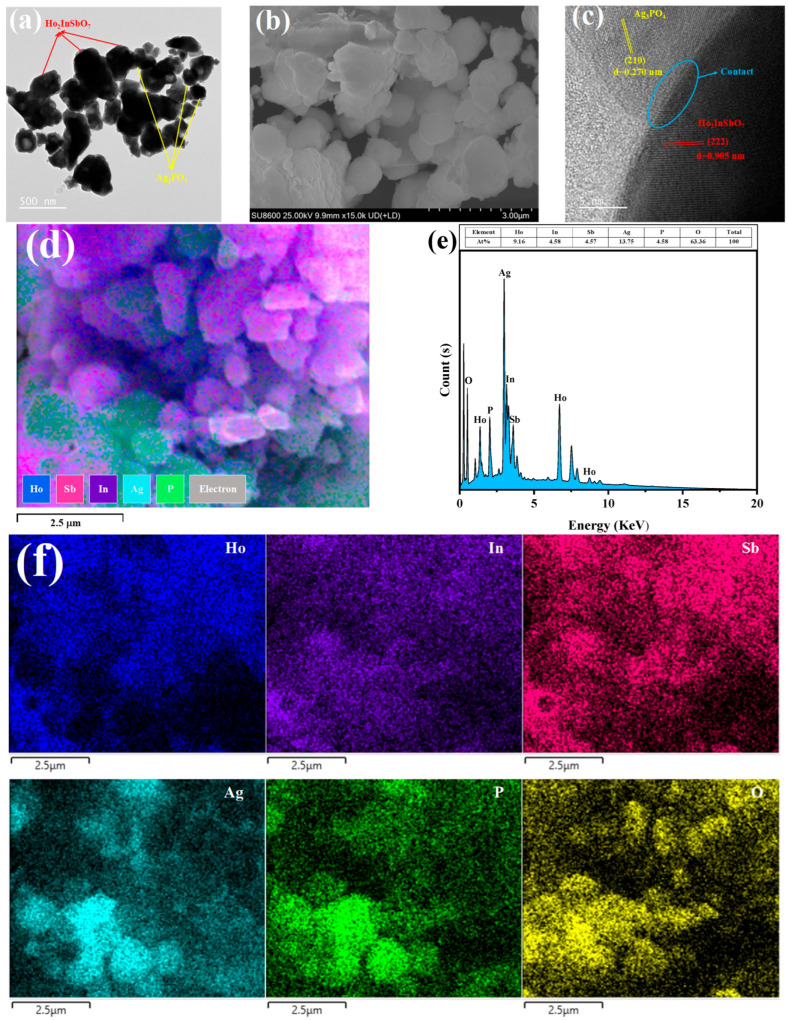
(**a**) TEM, (**b**) SEM, (**c**) HRTEM, (**d**) EDS layered, (**e**) EDS, and (**f**) EDS elemental mapping images of HAO.

In order to investigate the microstructural characteristics and morphology of the HAO heterojunction, a comprehensive analysis was performed utilizing TEM and SEM. Meanwhile, EDS was employed to analyze the elemental composition of the HAO heterojunction. Figure 2a,b present the TEM and SEM images of HAO heterojunction, clearly illustrating the coexistence of Ho_2_InSbO_7_ nanoparticles and Ag_3_PO_4_ nanoparticles within the heterojunction. Figure 2c showcases high-resolution TEM (HRTEM) images, enabling the clear identification of the interface structure between Ho_2_InSbO_7_ and Ag_3_PO_4_, as well as the lattice fringes of the two phases. This observation highlights the crucial role of the interface in promoting effective charge transfer among the constituent components.

Calculations revealed that the lattice spacing yielded 0.905 nm for the (222) plane of Ho_2_InSbO_7_, while the lattice fringes of the (210) crystal plane for Ag_3_PO_4_ are around 0.270 nm. The elemental distribution in the HAO heterostructure was investigated using the mapping shown in Figure 2d–f. The EDS elemental distribution analysis presented in Figure 2f indicates that the elements Ho, In, Sb, Ag, P, and O exhibit a homogeneous distribution within HAO heterojunction, providing direct evidence for the coexistence of the two phases, Ho_2_InSbO_7_ and Ag_3_PO_4_. A comparative analysis of the luminescence intensity between the regions corresponding to Ho, In, and Sb versus those corresponding to Ag and P suggests that Ho_2_InSbO_7_ predominantly exists as larger-sized particles, while Ag_3_PO_4_ is present in smaller-sized particles. Moreover, the comprehensive characterization results indicate that the preparation parameters established in this study significantly enhance the synthesis of high-purity HAO heterojunction photocatalytic materials.

**Figure 3:**
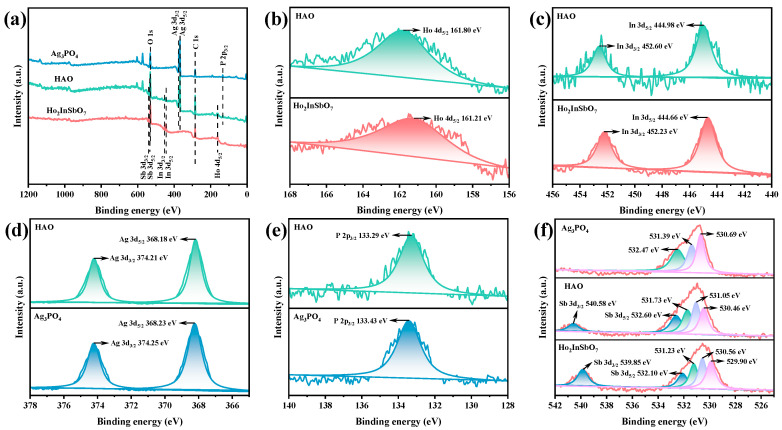
The XPS spectrum of synthesized Ho_2_InSbO_7_, Ag_3_PO_4_, and HAO: (**a**) survey spectrum, (**b**–**f**) high-resolution spectra of Ho 4d, In 3d, Ag 3d, P 2p, Sb 3d, and O 1s, respectively.

XPS was utilized to systematically analyze the elemental composition and chemical states of the HAO heterojunction photocatalyst, using Ho_2_InSbO_7_ and Ag_3_PO_4_ as references. The full-spectrum analysis presented in Figure 3a confirms the presence of Ho, In, Sb, Ag, P, and O elements within the HAO heterojunction, with each element originating from Ho_2_InSbO_7_ and Ag_3_PO_4_.

In Figure 3b, the Ho 4d_5_/_2_ peak shifts from 161.21 eV in Ho_2_InSbO_7_ to 161.80 eV in the HAO heterojunction. Figure 3c illustrates the In 3d orbital analysis, where the In 3d_3_/_2_ and In 3d_5_/_2_ peaks in Ho_2_InSbO_7_ are observed at 452.23 eV and 444.66 eV, respectively, with a spin–orbit splitting energy of 7.57 eV, indicating that the In element is in a +3 oxidation state [61,62,63]. In the heterojunction, the corresponding peaks shift to 452.60 eV and 444.98 eV. Figure 3d presents the Ag 3d orbital analysis, revealing that the Ag 3d_3/2_ and Ag 3d_5/2_ peaks in Ag_3_PO_4_ located at 374.25 eV and 368.23 eV, respectively, while in the HAO heterojunction, they shift to 374.21 eV and 368.18 eV. Figure 3e shows the P 2p orbital analysis, where the P 2p_3/2_ peak in Ag_3_PO_4_ is found at 133.43 eV, while in the heterojunction, the peak shifts to 133.29 eV. Finally, Figure 3f details the Sb 3d orbital analysis, indicating that the Sb 3d_3/2_ and Sb 3d_5/2_ peaks in Ho_2_InSbO_7_ located at 539.85 eV and 532.10 eV, respectively, with these peaks shifting to 540.58 eV and 532.60 eV in HAO heterojunction.

It is noteworthy that, compared to Ho_2_InSbO_7_, the binding energies of the Ho 4d, In 3d, and Sb 3d orbitals in the HAO heterojunction exhibit a positive shift. Conversely, when compared to Ag_3_PO_4_, the binding energies of the Ag 3d and P 2p orbitals show a negative shift. This characteristic displacement indicates a significant rearrangement of the electronic density within the heterojunction [64,65], specifically manifested by an increase in the electronic density surrounding the Ag and P, alongside a decrease in the electronic density around the Ho, In, and Sb. This phenomenon robustly confirms the successful construction of the HAO heterojunction and elucidates the mechanisms by which internal electronic interactions regulate the functional properties of the material.

The O 1s spectra of the HAO heterojunction, Ho_2_InSbO_7_, and Ag_3_PO_4_ were analyzed using peak fitting, as illustrated in Figure 3f. The peaks identified at 530.69 eV, 530.46 eV, and 529.90 eV are related to lattice oxygen [66]. Peaks found at 531.39 eV, 531.05 eV, and 530.56 eV are attributed to oxygen species that have been absorbed [66]. Moreover, the peaks at 532.47 eV, 531.73 eV, and 531.23 eV are linked to hydroxyl groups located on the surface [67].

Elemental surface composition analysis revealed that the HAO heterojunction exhibits an average atomic ratio of Ho/In/Sb/Ag/P/O = 853:426:428:1278:425:6590, showing a high degree of consistency with the findings from EDS. Notably, the atomic ratios of Ho/In/Sb and Ag/P are determined to be 2.00:1.00:1.00 and 3.01:1.00, respectively, which are consistent with the theoretical stoichiometric ratios. Importantly, no characteristic peaks indicative of secondary phases were detected in the XPS spectra.

These XPS findings provide critical experimental evidence to support the understanding of how the Z-scheme heterostructure is formed within the HAO heterojunction, while also revealing strong chemical interactions between Ho_2_InSbO_7_ and Ag_3_PO_4_ at the electronic structure level. This conclusion was corroborated by complementary characterization methods including XRD, Raman spectroscopy, FTIR, TEM, EDS, and SEM, collectively validates the successful synthesis of the heterojunction catalyst and elucidates the relationship between its structure and performance.

**Figure 4:**
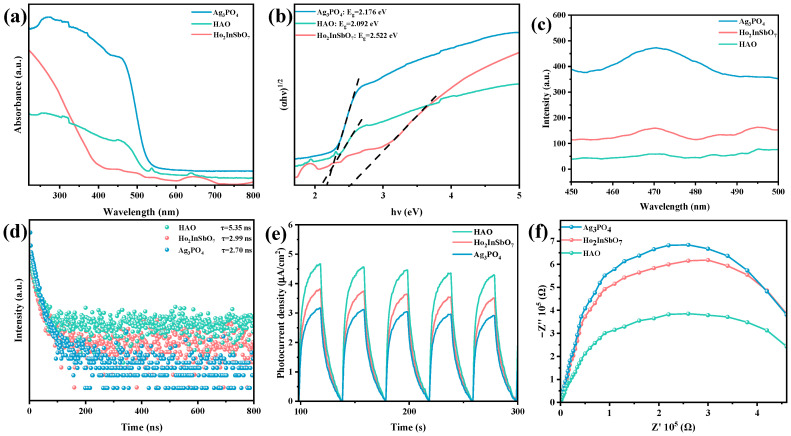
(**a**) UV–vis DRS and (**b**) corresponding plots of (*αhν*)^1/2^ and *hν* for Ho_2_InSbO_7_, Ag_3_PO_4_, and HAO; (**c**) PL spectra, (**d**) TRPL spectra, (**e**) PC curves, and (**f**) EIS plots of Ho_2_InSbO_7_, Ag_3_PO_4_, and HAO.

To further explore the band structure properties of the synthesized catalysts, this study systematically analyzed the UV-Vis DRS of Ho_2_InSbO_7_, Ag_3_PO_4_, and the HAO heterojunction, as shown in Figure 4a. The absorption onset for Ho_2_InSbO_7_ was recorded at 512 nm, whereas Ag_3_PO_4_ exhibited an absorption edge at 550 nm. In contrast, the HAO heterojunction displayed a notable red shift in its absorption edge, reaching 570 nm, indicative of a broader spectral response. This phenomenon highlights the enhanced light absorption capability of the HAO heterojunction compared to the individual component materials.

The band gap of the semiconductor materials could be precisely determined based on the Kubelka–Munk function, as illustrated in Equation (1) [68,69]. By calculating the tangent of the linear region of the diffuse reflectance absorption spectrum and extrapolating it to the *x*-axis intersection, the band gap width could be accurately ascertained.

(1) $$\frac{{\left[1-R_{d}(h\nu )\right]}^{2}}{2R_{d}(h\nu )}=\frac{\alpha (h\nu )}{S}$$

In this function, the absorption coefficient, scattering coefficient, and diffuse reflectance are denoted by *α*, *S*, and *R_d_*, respectively. Furthermore, the optical absorption characteristics of the material near the band edge adhere to the mathematical relationship described in Equation (2) [70,71,72]:

(2) (αhν)1n = A(hν−Eg)

In the equation, the proportionality constant is denoted by *A*, the absorption coefficient by *α*, the bandgap energy by *E_g_*, and the photon frequency by *ν*. The parameter *n*, which characterizes the nature of electronic transitions induced by optical excitation, takes the value 1/2 for direct transitions and 2 for indirect transitions [72]. The bandgap energies of Ho_2_InSbO_7_ and Ag_3_PO_4_ were calculated as 2.522 eV and 2.176 eV, respectively, through the analysis of the data presented in Figure 4b. Both materials exhibit n values approaching 2, suggesting that their interband transitions are of the indirect type. The calculated bandgap energy of HAO heterojunction was determined to be 2.092 eV, which also suggests the presence of indirect transition behavior. The significantly reduced bandgap width corroborates the enhanced optical absorption performance of this heterojunction.

To evaluate the photocatalytic degradation efficiency and the recombination rate of PGCs of the prepared photocatalysts, PL spectroscopy was conducted in this study. The intensity of photoluminescence is positively correlated with the rate of carrier recombination; thus, stronger luminescent signals indicate more significant recombination processes, while weaker luminescent intensities reflect enhanced charge carrier migration efficiencies [73,74]. As illustrated in Figure 4c, the HAO heterojunction, Ho_2_InSbO_7_, and Ag_3_PO_4_ all exhibit broad emission peaks around 470 nm. Notably, HAO heterojunction demonstrates the lowest photoluminescence intensity, suggesting that it possesses the most effective charge separation efficiency and capacity to inhibit the recombination of PGCs. In comparison, Ho_2_InSbO_7_ exhibits a higher photoluminescence intensity, while Ag_3_PO_4_ displays the strongest luminescent signal. This phenomenon provides strong evidence, from a photophysical perspective, that the HAO heterojunction demonstrates superior photocatalytic performance in the degradation of OTC.

To further validate this conclusion, TRPL spectroscopy was used to measure the carrier lifetime in Ho_2_InSbO_7_, Ag_3_PO_4_, and HAO heterojunction. The results are presented in Figure 4d. The average photogenerated carrier lifetime ($\tau_{ave}$) can be calculated using Formula (3) [75,76,77]:

(3) $$\tau_{ave}=(A_{1}\tau_{1}^{2}+A_{2}\tau_{2}^{2})/(A_{1}\tau_{1}+A_{2}\tau_{2})$$

The formation of heterojunctions enhances the separation of PGCs. Consequently, HAO heterojunction exhibits a significantly prolonged carrier lifetime of ($\tau_{ave}$ = 5.35 ns), in comparison to Ho_2_InSbO_7_ ($\tau_{ave}$ = 2.99 ns) and Ag_3_PO_4_ ($\tau_{ave}$ = 2.70 ns). The relatively short carrier lifetimes observed for Ho_2_InSbO_7_ and Ag_3_PO_4_ suggest a more pronounced recombination of PGCs. The extended carrier lifetime in HAO heterojunction may stem from the effective separation of carriers at the heterojunction interface. This finding is highly consistent with the conclusions drawn from the PL spectroscopy analysis, providing strong evidence of the superior ability of HAO heterojunction in regulating photogenerated carrier lifetimes.

Figure 4e,f shows the PC responses and EIS recorded for the samples to evaluate the charge carrier separation efficiency and interfacial charge transfer behavior of HAO heterojunction, Ho_2_InSbO_7_, and Ag_3_PO_4_. As illustrated in Figure 4e, the HAO heterojunction exhibits a significantly enhanced photocurrent density, which is substantially higher than that of the individual component materials. This phenomenon indicates that the composite structure of Ho_2_InSbO_7_ and Ag_3_PO_4_ significantly enhances the separation efficiency of PGCs [78,79]. The sustained and stable photocurrent signal reflects the outstanding photocatalytic stability of the synthesized catalyst. Figure 4f presents the EIS spectra of the prepared photocatalysts. A smaller semicircle radius generally corresponds to a lower charge transfer resistance [80,81]. The semicircle radius of HAO heterojunction is notably smaller than that of the individual component materials, confirming its enhanced charge separation capability. This result is consistent with findings from PL spectra, TRPL spectra, and PC tests. The aforementioned analysis demonstrates that the heterojunction structure formed by Ho_2_InSbO_7_ and Ag_3_PO_4_ significantly enhances the separation efficiency of photoinduced electron-hole pairs, thus extending the carrier lifespan and providing critical mechanistic support for the optimization of photocatalytic performance.

### 2.2. Examination of Photocatalytic Efficiency

#### 2.2.1. Photocatalytic Degradation of OTC

**Figure 5:**
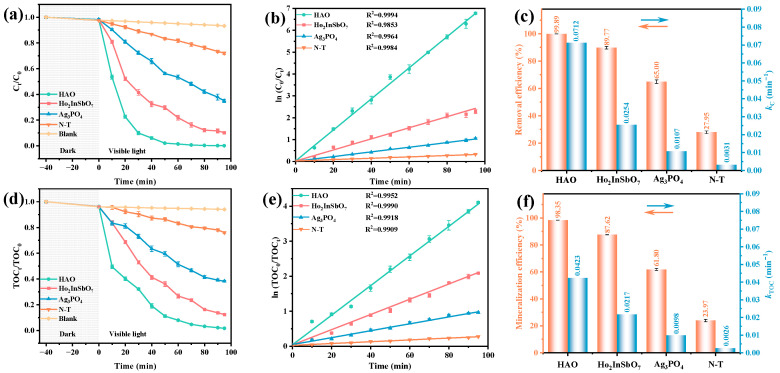
(**a**) Photodegradation, (**b**) kinetic curves, and (**c**) removal efficiencies and kinetic constants for OTC; (**d**) mineralization, (**e**) kinetic curves, and (**f**) mineralization efficiencies and kinetic constants for TOC.

A comprehensive evaluation was conducted to validate the photocatalytic degradation performance of the HAO heterojunction, Ho_2_InSbO_7_, Ag_3_PO_4_, and nitrogen-doped titanium dioxide (N-T) under VLI for the degradation of OTC in antibiotic wastewater. Among them, N-T, as a well-recognized visible-light responsive photocatalyst, was used as a benchmark to evaluate and compare the differences in photodegradation efficiency among different catalyst samples. As illustrated in Figure 5a, all experimental groups containing catalysts exhibited a significant decrease in OTC concentration, whereas the blank control group (photolysis without a catalyst) showed no noticeable change in OTC concentration. This finding confirms the dominant role of photocatalysts in the photocatalytic degradation process. Figure 5b presents the first-order kinetic constants (*k*_C_) for the photocatalytic degradation of OTC by the HAO heterojunction, Ho_2_InSbO_7_, Ag_3_PO_4_, and N-T under VLI. The kinetic parameters were derived from the linear fitting of the photodegradation time-concentration curves, based on the kinetic equation, ($ln(C_{0}/C_{t})=k_{C}t)$ where *C*_t_ and *C*_0_ represent the instantaneous concentration and the initial concentration of OTC, respectively. The kinetically determined *k*_C_ for the degradation of OTC utilizing the HAO heterojunction reached 0.0712 min^−1^, representing 2.80-, 6.65-, and 22.97-fold improvements over Ho_2_InSbO_7_, Ag_3_PO_4_, and N-T, respectively. The PRE was determined by the formula ($1-C_{t}/C_{0}$) × 100%, where *C*_t_ and *C*_0_ represent the instantaneous concentration and the starting concentration of OTC, respectively. The results presented in Figure 5c indicate that the HAO heterojunction, when employed as a photocatalyst, attained an outstanding OTC PRE of 99.89% following 95 min of VLI. In comparison, the OTC PRE using Ho_2_InSbO_7_ and Ag_3_PO_4_ as photocatalysts were 89.77% and 65.00%, respectively. Conversely, the N-T photocatalyst demonstrated a significantly lower PRE of only 27.95%. Notably, the HAO heterojunction exhibited enhancements in OTC removal rates of 1.11 times, 1.54 times, and 3.57 times greater than those achieved with Ho_2_InSbO_7_, Ag_3_PO_4_, and N-T, respectively, thereby highlighting its exceptional catalytic performance. Figure 5d further illustrates the mineralization effects on total organic carbon (TOC) under VLI conditions for the various photocatalysts employed. Consistent with the degradation results for OTC, the prepared catalysts exhibited notable TOC mineralization efficiencies. Notably, the HAO heterojunction demonstrated exceptional performance, yielding mineralization efficiencies in the order of HAO > Ho_2_InSbO_7_ > Ag_3_PO_4_ > N-T. Figure 5e displays the *k*_C_ calculated for the photocatalytic degradation of TOC by the HAO heterojunction, Ho_2_InSbO_7_, Ag_3_PO_4_, and N-T under VLI conditions, based on the kinetic equations (where *TOC*_t_ and *TOC*_0_ denote the instantaneous and starting concentrations of TOC, respectively). As shown in Figure 5f, the results of the kinetic constant (*k*_TOC_) measurements reveal that the mineralization kinetic efficiency of the HAO heterojunction (0.0423 min^−1^) is 1.95 times that of Ho_2_InSbO_7_ (0.0217 min^−1^), 4.32 times that of Ag_3_PO_4_ (0.0098 min^−1^), and 16.27 times that of N-T (0.0026 min^−1^). Notably, all catalysts showed *k*_TOC_ values lower than *k*_C_ values, revealing the emergence and accumulation of intermediate species during the photocatalytic degradation process. The mineralization rate of TOC can be calculated using the formula: ($ln{(TOC}_{0}/{TOC}_{t})=k_{TOC}t)$, where *TOC*_0_ and *TOC*_t_ denote the initial and instantaneous concentrations of TOC, respectively. The results indicate that, after 95 min of VLI, the HAO heterojunction achieved a mineralization efficiency of 98.35% for TOC, which is significantly higher than that of Ho_2_InSbO_7_ (87.62%), Ag_3_PO_4_ (61.80%), and N-T (23.97%). Comparative analysis reveals that the mineralization rate of TOC for the HAO heterojunction is enhanced by factors of 1.12, 1.59, and 4.10 times relative to Ho_2_InSbO_7_, Ag_3_PO_4_, and N-T, respectively, thereby corroborating its superior ability for the deep mineralization of organic pollutants. Collectively, these findings demonstrate that the HAO heterojunction photocatalyst exhibits significant advantages both in terms of OTC degradation efficiency and TOC mineralization extent, providing a novel solution for the efficient treatment of antibiotic-laden wastewater.

**Figure 6:**
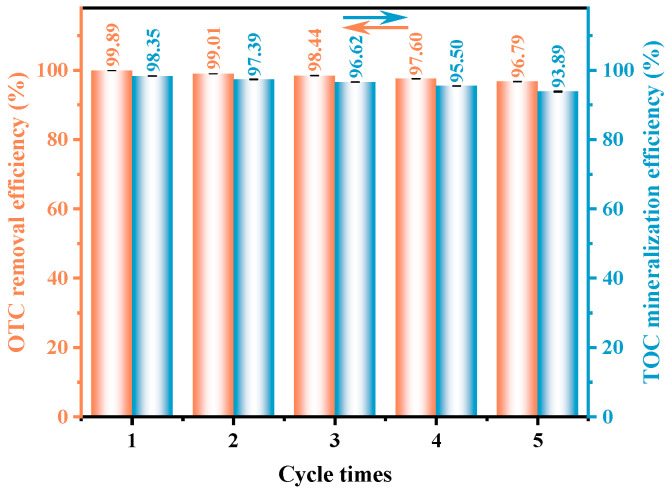
Five consecutive tests on HAO for the degradation of OTC and the mineralization of TOC under VLI.

The stability and durability of the synthesized HAO heterojunction photocatalyst were assessed through a set of cyclic experiments, with the outcomes displayed in Figure 6 and Figure S3. Notably, during five consecutive cycles of OTC degradation under VLI, the removal rate of OTC decreased by only 3.10%, while the mineralization rate of TOC showed a reduction of merely 4.46%. These findings clearly demonstrate the exceptional structural stability and reusability potential of the fabricated photocatalytic material, highlighting its feasibility for practical applications in wastewater treatment. In addition, to systematically evaluate the structural stability of HAO, XRD and SEM analyses were performed on both fresh and post-reaction samples. As illustrated in Figure S4, the characteristic diffraction peaks of the used HAO sample are nearly identical to those of the fresh sample. No additional diffraction peaks were observed in the XRD pattern after prolonged photocatalytic degradation, and the SEM images revealed no significant changes in particle morphology. These characterization results collectively demonstrate that the heterojunction structure maintains excellent structural stability under operational conditions.

In addition, Figure S5 illustrates the influence of varying HAO dosages on the degradation efficiency of OTC under VLI over a 95 min period. The highest PRE of OTC, reaching 99.89%, was observed when the initial HAO concentration was 0.6 g/L. However, further increases in HAO dosage resulted in a decline in OTC degradation efficiency. This reduction could be attributed to the aggregation of HAO at higher concentrations, which limits the availability of active sites on the catalyst surface.

Figure S6 presents the impact of different pH values on OTC degradation efficiency when HAO is employed as a catalyst under VLI. Notably, after 95 min of irradiation, OTC exhibited consistently high degradation efficiencies across all tested pH levels (3, 7, and 11). Specifically, the PRE were measured at 99.84%, 99.89%, and 98.87% for pH 3, 7, and 11, respectively. These results indicate that pH variation has minimal influence on OTC degradation efficiency under visible light when HAO is used as the photocatalyst.

**Figure 7:**
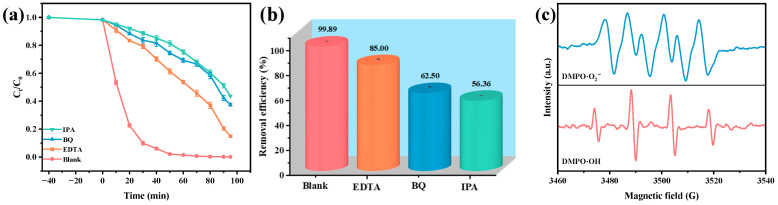
Impact of different radical scavengers on (**a**) OTC saturation, (**b**) removal efficiency of OTC, and (**c**) EPR spectrum for DMPO•O_2_^−^ and DMPO•OH over HAO.

To elucidate the mechanism of active radical involvement in the photocatalytic degradation of OTC by the HAO heterojunction, this study conducted a series of radical scavenging experiments. Ethylenediaminetetraacetic acid (EDTA), benzoquinone (BQ), and isopropanol (IPA) were employed as selective scavengers for h^+^, •O_2_^−^, and •OH, respectively. The experiments revealed that the degradation efficiency of OTC reached an impressive 99.89% in the absence of any scavengers in Figure 7a,b. However, upon the introduction of EDTA, BQ, and IPA, the degradation efficiencies significantly decreased to 85.00%, 62.50%, and 56.36%, respectively. These results indicate the dominant role of •OH radicals in the photocatalytic degradation mechanism. EPR spectroscopy was employed to further investigate the production behavior of reactive free radicals, specifically •O_2_^−^ and •OH, during the photocatalytic degradation of OTC by the HAO heterojunction photocatalyst. Figure 7c depicts the distinct EPR signal characteristics of the DMPO·OH and DMPO•O_2_^−^ adducts within the HAO heterojunction system. Notably, after 10 min of VLI, the spectrum of EPR revealed a four-peak pattern with equivalent intensity (1:1:1:1 ratio), consistent with the signal characteristics of DMPO•O_2_^−^, thus confirming the presence of the •O_2_^−^ radical [79]. Additionally, a characteristic 1:2:2:1 splitting pattern in the DMPO•OH adduct was identified by EPR, furnishing critical evidence for •OH radical generation during VLI-driven photocatalysis [82]. Importantly, analysis of the relative signal intensities revealed that the quantity of •OH generated was significantly greater than that of •O_2_^−^. Notably, this finding is consistent with radical trapping experiment data, furnishing direct evidence for the synergistic contribution of •O_2_^−^ and •OH radicals to degradation reactions [82,83].

#### 2.2.2. Comparison of Photocatalytic Activity

Our study highlights the distinctive advantages of HAO through a comprehensive comparative analysis. Table 1 provides a systematic overview of existing research in this field, demonstrating the remarkably high efficiency of OTC degradation achieved by employing HAO as a photocatalyst. The results clearly indicate that HAO outperforms other catalysts in photocatalytic efficacy, thereby revealing its considerable potential. These findings not only elucidate the pivotal role of HAO in accelerating OTC photodegradation but also constitute a significant contribution to the broader domain of photocatalysis, further affirming its relevance and importance.

#### 2.2.3. Possible Photocatalytic Mechanism of HAO

**Figure 8:**
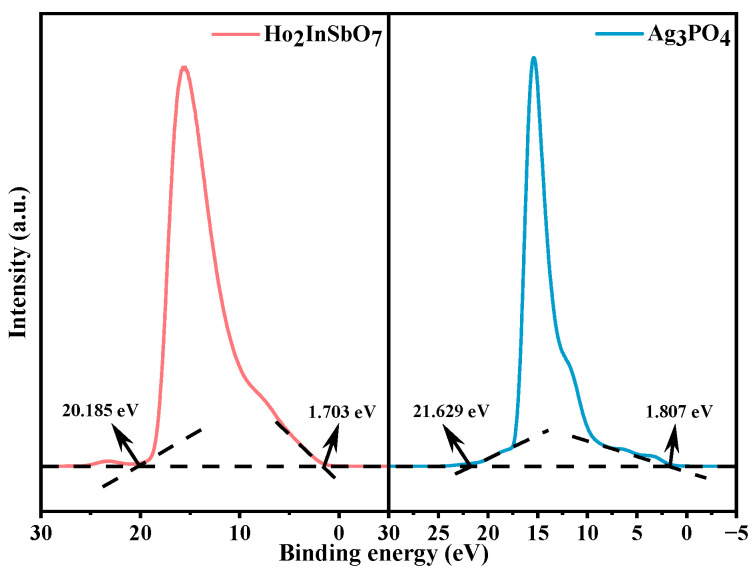
UPS spectra of Ho_2_InSbO_7_ and Ag_3_PO_4_ (the intersections of the black dash lines indicated by the black arrows indicated the onset (Ei) and cutoff (Ecutoff) binding energy).

To elucidate the energy band structure characteristics of the HAO heterojunction, this study systematically characterized the ionization potentials of its constituent materials using UPS. As shown in Figure 8, the UPS measurements indicate that the initial binding energy and the cutoff binding energy for Ho_2_InSbO_7_ are 1.703 eV and 20.185 eV, respectively, while for Ag_3_PO_4_, the corresponding values are 1.807 eV and 21.629 eV [90]. The ionization potentials of Ho_2_InSbO_7_ (2.718 eV) and Ag_3_PO_4_ (1.378 eV) were determined using an excitation photon energy of 21.2 eV [91]. On the basis of the ionization potential data and the diffuse reflectance absorption spectroscopy results, further analysis reveals that the conduction band (CB) edge of Ho_2_InSbO_7_ is situated at 0.068 eV, while that of Ag_3_PO_4_ is located at −0.802 eV. This energy level discrepancy provides critical theoretical insight into the directional migration behavior of charge carriers within the heterojunction system.

**Figure 9:**
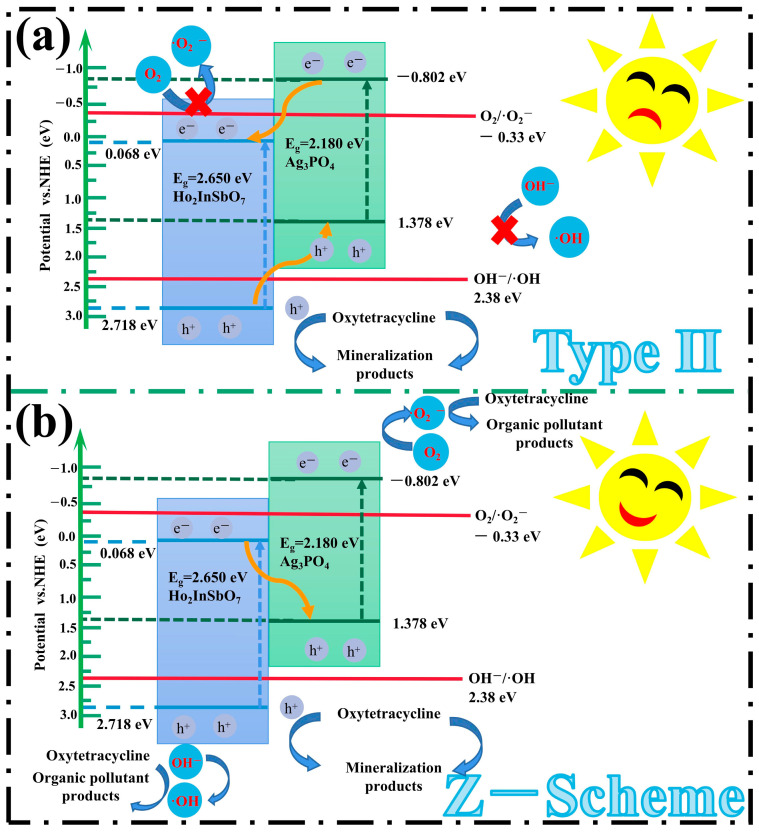
Plausible photodegradation mechanism of OTC with HAO as photocatalyst under VLI: (**a**) type Ⅱ and (**b**) Z-Scheme.

Based on the band structure characteristics of Ho_2_InSbO_7_ and Ag_3_PO_4_, this study thoroughly investigates the photocatalytic reaction mechanisms of the HAO heterojunction. As illustrated in Figure 9, two potential charge transfer pathways are proposed: Type II and Z-scheme. In the conventional Type II heterojunction structure, it is hypothesized that the photoinduced electrons generated from the CB of Ag_3_PO_4_ migrate to the CB of Ho_2_InSbO_7_, whereas holes derived from the valence band (VB) of Ho_2_InSbO_7_ move to the VB of Ag_3_PO_4_. However, this model presents theoretical inconsistencies. Specifically, the CB potential of Ho_2_InSbO_7_ (*E*_CB_ = 0.068 eV) exceeds the reduction potential of the O_2_/•O_2_^−^ couple (−0.33 eV vs. NHE) [92], which renders its CB electrons unable to effectively reduce O_2_ into •O_2_^−^. Concurrently, the VB potential of Ag_3_PO_4_ (*E*_VB_ = 1.378 eV) is lower than the oxidation potential of the OH^−^/•OH couple (2.38 eV vs. NHE) [93,94], thereby preventing the holes from oxidizing OH^−^ to generate •OH. These conclusions conflict with the findings from radical scavenging assays and EPR analysis, highlighting significant discrepancies in the proposed mechanism.

In the Z-scheme heterojunction, photogenerated electrons migrate from the CB of Ho_2_InSbO_7_ (0.068 eV) to the VB of Ag_3_PO_4_ (1.378 eV), thereby promoting the recombination of carriers with lower redox potential within the heterojunction. This process preserves high-energy carriers, thus improving the overall catalytic performance. Specifically, the electrons in the CB of Ag_3_PO_4_ (−0.802 eV) could effectively participate in reactions with O_2_ to generate •O_2_^−^, while the h^+^ in the VB of Ho_2_InSbO_7_ (2.718 eV) could oxidize OH^−^ to generate •OH. This reaction pathway elucidates the synergistic interactions between the two materials, contributing to the observed photocatalytic enhancement. Furthermore, the h^+^ present in the VB of Ho_2_InSbO_7_ and Ag_3_PO_4_ could directly engage in the oxidative degradation of OTC due to their strong oxidizing capability. The synergistic actions of the generated reactive radicals, including •O_2_^−^, •OH, and h^+^, are pivotal for pollutant degradation, which is highly consistent with the results from radical scavenging assays and EPR characterizations. The mechanistic insights provided not only elucidate the photocatalytic reaction pathways of the HAO heterojunction but also highlight its substantial advantages in the field of environmental remediation. This research paves the way for theoretical advancements in the efficient photocatalytic degradation of OTC.

#### 2.2.4. Possible Degradation Pathway of OTC

**Figure 10:**
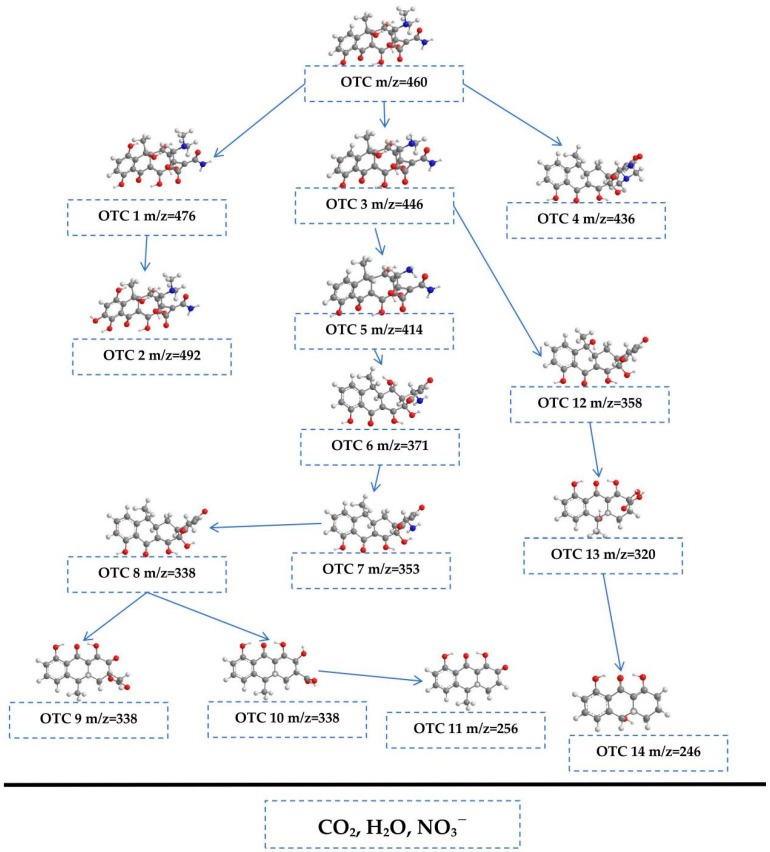
Viable photodegradation pathway for OTC under VE with HAO heterojunction as catalyst.

The potential pathways for the photodegradation of OTC are illustrated in Figure 10. The degradation process encompasses several key reactions, including hydroxylation, demethylation, dehydration, decarboxamidation, and ring-opening. The ion detected at a mass-to-charge ratio (*m*/*z*) of 460 represents the OTC molecule [95]. Within the photocatalytic system, reactive •OH may target the aromatic ring of OTC, resulting in the formation of two hydroxylated products: OTC 1 (*m*/*z* = 476) and OTC 2 (*m*/*z* = 492) [95]. Furthermore, OTC 3 (*m*/*z* = 446) could result from the demethylation process, where the N-methyl group is removed [95]. OTC 4 (*m*/*z* = 426) was generated from OTC through a dehydration reaction, which involved the loss of a water molecule [95]. OTC 3 could follow two distinct degradation pathways. The first pathway involved its transformation into OTC 5 (*m*/*z* = 414) via the simultaneous loss of both the N-methyl and hydroxyl groups [95]. Subsequently, OTC 5 could undergo further decomposition to yield OTC 6 (*m*/*z* = 371) through a decarboxamidation process [95]. OTC 7 (*m*/*z* = 353) may be derived from OTC 6 through another dehydration route, whereas OTC 8 (*m*/*z* = 338) is produced from OTC 7 by means of a deamination reaction [95]. These reactions illustrated the complex transformations that OTC could undergo during photocatalytic degradation, revealing the intricate mechanisms at play in the remediation process. Following these reactions, •OH and •O_2_^−^ could attack the double bond present in OTC 8, facilitating the formation of OTC 9 (*m*/*z* = 312) and OTC 10 (*m*/*z* = 302) through ring-opening mechanisms [95]. Subsequently, OTC 10 may undergo further transformation into OTC 11 (*m*/*z* = 256) through decarboxylation and dehydration processes [95]. Additionally, OTC 3 could fragment, leading to the generation of OTC 12 (*m*/*z* = 358) through the elimination of N-methyl, amide, hydroxyl groups, and carboxamide [95]. Ring-opening of OTC 12 may then occur, leading to the formation of OTC 13 (*m*/*z* = 320). This compound could then decompose into OTC 14 (*m*/*z* = 246) via a series of dehydration, decarboxylation, and demethylation reactions [95]. During the course of this degradation pathway, intermediates OTC 11 and OTC 14 underwent further transformations involving ring-opening, dehydration, and demethylation reactions. These transformations may lead to the oxidation of intermediates, generating a variety of byproducts. Ultimately, the degradation pathway culminates in the formation of small organic molecules, many of which undergo further photocatalytic oxidation, resulting in the conversion of these compounds into carbon water (H_2_O), dioxide (CO_2_), and nitrate ions (NO_3_^−^).

## 3. Experimental Section

### 3.1. Materials and Reagents

All chemical reagents were used directly without further purification. Commonly used reagents utilized in this research were procured from established suppliers to ensure high purity and reliability. Specifically, the subsequent chemicals were obtained from Macklin Biochemical Co., Ltd., Shanghai, China: SbCl_5_ (purity = 99.999%), In(NO_3_)_3_·5H_2_O (purity = 99.99%), Ho(NO_3_)_3_·5H_2_O (purity = 99.99%), and benzoquinone (BQ, C_6_H_4_O_2_, purity ≥ 99.5%). Moreover, pure ethanol (C_2_H_5_OH, purity ≥ 99.5%) and ethylenediaminetetraacetic acid (EDTA, C_10_H_16_N_2_O_8_, purity = 99.995%) were sourced from Merck Co., Ltd., Shanghai, China. Furthermore, Ag_3_PO_4_ (purity > 98%), isopropyl alcohol (IPA, C_3_H_8_O, purity ≥ 99.999%), octanol (C_8_H_18_O, purity ≥ 99.5%), and oxytetracycline (OTC, C_22_H_24_N_2_O_9_, purity ≥ 98%) were obtained from Aladdin Group Chemical Reagent Co., Ltd., Shanghai, China.

### 3.2. Synthesis of N-Doped TiO_2_

Characterization details are provided in the Supplementary Materials (Section S1).

### 3.3. Preparation Method of Ho_2_InSbO_7_

In this study, we synthesized the catalyst Ho_2_InSbO_7_ employing a solvothermal method. The synthesis commenced with the careful preparation of precursor solutions, consisting of equal volumes of Ho(NO_3_)_3_·5H_2_O (0.34 mol/L), In(NO_3_)_3_·5H_2_O (0.17 mol/L), and SbCl_5_ (0.17 mol/L). These components were thoroughly mixed and subjected to magnetic stirring for a duration of 1420 min to ensure homogeneity. Following this, the uniform precursor solution was placed into a PTFE-lined high-pressure reactor. The temperature within the autoclave was subsequently held constant at 215 °C for a duration of 790 min, facilitating the initial formation of the desired compound. Subsequently, the mixture underwent further thermal treatment in a tube nitrogen-rich furnace environment. A controlled rate of 4.8 °C/min was used to incrementally increase the temperature until it reached 280 °C, which was then held for an additional 540 min. This synthesis process culminated in the successful preparation of pure Ho_2_InSbO_7_ powder.

### 3.4. Synthesis of HAO Heterojunction Photocatalyst

The synthesis of the HAO heterostructure catalyst was achieved via an ultrasound-assisted solvothermal method. Initially, equimolar amounts of Ho_2_InSbO_7_ and Ag_3_PO_4_, both synthesized through prior solvothermal processes, were combined in octanol. This mixture was sonicated for 275 min using an ultrasonic bath to promote uniform dispersion. Following sonication, the mixture underwent magnetic stirring at a steady temperature of 225 °C for a duration of 270 min. This heating stage enabled surface deposition of Ho_2_InSbO_7_ onto Ag_3_PO_4_ nanocrystals, giving rise to the HAO heterostructured catalyst. After the reaction was completed, room temperature cooling of the mixture was permitted, and the resulting product was separated by centrifugation. To ensure thorough purification, the product was washed multiple times with ethanol. Following purification, the powder was vacuum-dried in an oven at 130 °C for 110 min and then stored in a desiccator to prevent moisture absorption. Thus, the successful synthesis of the HAO heterostructure catalyst was accomplished.

### 3.5. Characterization

Characterization details are provided in the Supplementary Materials (Section S2).

### 3.6. Photoelectrochemical Experiments

For electrochemical characterization, the synthesized photocatalyst (5 mg) was dispersed in a mixed solvent comprising 450 µL of ethylene glycol and ethanol. The obtained suspension underwent ultrasonic processing for 45 min to achieve complete dispersion of the catalyst particles. The mixture was then stirred magnetically for a further 100 min to obtain a uniformly dispersed suspension. A 45 µL aliquot of the freshly made suspension was then applied onto a glassy carbon electrode through drop-casting and allowed to dry under ambient conditions for 50 min, forming a uniform photocatalytic film. Electrochemical tests were conducted employing a CHI-660D electrochemical workstation manufactured by Chenhua Instruments Co., Ltd. (Shanghai, China). The counter electrode was a platinum sheet, whereas the reference electrode was an Ag/AgCl electrode. Under light irradiation, a 0.12 mol/L Na_2_SO_4_ aqueous solution was used to record the transient photocurrent responses. In a 0.12 mol/L KCl solution, EIS was performed to further assess the electrochemical properties of the photocatalyst. These measurements provided insights into the charge transfer behavior and interface characteristics of the photocatalytic materials.

### 3.7. Experimental Setup and Procedure

To evaluate the adsorption and photodegradation activities of the photocatalysts (Ho_2_InSbO_7_, Ag_3_PO_4_, and HAO), a concentration of 0.6 g/L of each catalyst was introduced into 460 mL of a 10 mg/L OTC solution. The experiments were conducted in a photocatalytic reactor (CEL-LB70, China Education Au-Light Technology Co., Ltd., Beijing, China). An initial adsorption step was performed in the dark for 40 min to ensure even distribution of the photocatalysts and complete saturation of OTC adsorption.

Subsequently, the photodegradation reaction was started under visible light irradiation by employing a 500 W xenon lamp fitted with a 420 nm cutoff filter. Throughout the degradation procedure, 5 mL aliquots of the reaction mixture were withdrawn every 10 min for analysis. Each collected sample underwent centrifugation at 8000 revolutions per minute for a duration of 20 min to isolate the clear supernatant, which was subsequently used to determine the remaining OTC concentration. The residual OTC content was quantified using a high-performance liquid chromatography (HPLC) system (Agilent 200, Agilent Technologies, Palo Alto, CA, USA). To facilitate analysis, 10 microliters of the clarified supernatant was loaded into the HPLC apparatus with a flow rate set at 0.5 mL/min.

The mineralization extent of TOC during OTC degradation was assessed using a Shimadzu TOC-5000A analyzer (Kyoto, Japan). Potassium hydrogen phthalate (KHC_8_H_4_O_4_) served as the certified reference material for calibrating TOC measurements across the photodegradation process. Calibration solutions were prepared with carbon concentrations from 0 to 100 mg/L, using potassium hydrogen phthalate as the primary standard.

The quantification and identification of OTC and its photodegradation products were performed using liquid chromatography-mass spectrometry (LC-MS) on a Thermo Quest LCQ Duo system (Thermo Fisher Scientific, Waltham, MA, USA). Chromatographic separation was achieved using a Beta Basic-C18 HPLC column (150 × 2.1 mm, 5 μm particle size; Thermo Fisher Scientific) operated in positive electrospray ionization (ESI+) mode. The mass spectrometry parameters were optimized as follows: spray voltage, 4.0 kV; sheath gas pressure, 30 psi; auxiliary gas flow rate, 10 arbitrary units; capillary temperature, 350 °C; source CID collision energy, 0 V; and collision pressure, 1.5 mTorr. The collision energy for fragmentation was set to −38 eV. The mobile phase consisted of 0.1% (*v*/*v*) formic acid in water (solvent A) and acetonitrile (solvent B), delivered at a flow rate of 0.4 mL/min with an injection volume of 5 μL. A linear gradient elution program was employed: initial conditions of 90% A were linearly decreased to 10% A over 13 min, followed by an isocratic hold at 100% B for 5 min to ensure complete elution of retained compounds. The column temperature was maintained at 303 K (30 °C) throughout the analysis.

## 4. Conclusions

In conclusion, this study successfully fabricated a direct Z-scheme HAO heterojunction photocatalyst, utilizing an ultrasound-assisted solvothermal method. In comparison to pure Ho_2_InSbO_7_ and Ag_3_PO_4_, HAO demonstrated markedly enhanced photocatalytic performance in the degradation of OTC under VLI. Specifically, the OTC removal efficiency achieved by HAO was 99.89% within 95 min, indicating enhancements of 1.11, 1.54, and 3.57 times relative to Ho_2_InSbO_7_, Ag_3_PO_4_, and N-T, respectively. This substantial improvement could be attributed to the distinctive Z-scheme electron transfer mechanism facilitated by the interaction between Ho_2_InSbO_7_ and Ag_3_PO_4_, which significantly enhances both the separation efficiency of PGCs and the redox activity of HAO. Moreover, cycling experiments and supplementary characterizations corroborated the enhanced structural stability and adaptability of HAO. EPR analysis and quenching experiments further substantiated the crucial functions of •OH, •O_2_^−^, and h^+^ during the photocatalytic degradation of OTC. In addition, reliable pathways and mechanisms for the degradation of OTC were elucidated. Future research will explore loading HAO onto high-specific-surface-area materials to address low photogenerated charge carrier separation efficiency and insufficient active sites due to catalyst agglomeration, leveraging synergistic effects. In conclusion, this study demonstrates that the HAO heterojunction photocatalyst holds significant potential for remediating antibiotic-contaminated wastewater and offers a novel approach for degrading refractory organic pollutants, with substantial application value.

## Figures and Tables

**Table 1:** Comparison of photocatalytic efficacy of HAO with other reported photocatalysts in photodegradation process of OTC.

Photocatalyst	Radiation	Irradiation Time (min)	Antibiotic	Removal Rate (%)	Ref.
N_i_C_o_/Z_n_O/_g_-C_3_N_4_	Visible light	50	Oxytetracycline	71.3	[84]
B_i_VO_4_	sunlight conditions	240	Oxytetracycline	83	[85]
CuBi_2_O_4_/BiVO_4_	Visible light	60	Oxytetracycline	81.11	[86]
NiO/BiOBr	Visible light	120	Oxytetracycline	72.6	[87]
MoS_2_/UiO-66	simulated sunlight	120	Oxytetracycline	86.6	[88]
In_2_S_3_/Gd_2_O_3_	Visible light	50	Oxytetracycline	80	[89]
Ho_2_InSbO_7_	Visible light	95	Oxytetracycline	89.77	This study
HAO	Visible light	95	Oxytetracycline	99.89	This study

## Data Availability

Data are contained within the article.

## Supplementary Materials

The following supporting information can be downloaded at: https://www.mdpi.com/article/10.3390/molecules30153289/s1, Figure S1. (a) XRD pattern and Rietveld refinement and (b) the atomic architecture (red atom: O; green atom: Ho; purple atom: In or Sb) of Ho_2_InSbO_7_. Figure S2. (a) XRD pattern and Rietveld refinement and (b) the atomic architecture (red atom: O; blue atom: P; pink atom: Ag) of Ag_3_PO_4_. Figure S3. (a) Photodegradation; (b) kinetic curves, and (c) removal efficiencies and kinetic constants for five consecutive OTC degradation tests; (d) mineralization, (e) kinetic curves, and (f) mineralization efficiencies and kinetic constants for five consecutive TOC mineralization tests. Table S1. Configurable properties of Ho_2_InSbO_7_ fabricated using solvothermal method. Table S2. Configurable properties of Ag_3_PO_4_ fabricated using solvothermal method. Section S1. Synthesis of N-Doped TiO_2_. Section S2. Characterization. Figure S4. (a) XRD and (b) SEM patterns of the fresh and the used HAO. Figure S5. Impact of HAO dosage on removal efficiency of OTC. Figure S6. The effect of different pH values on OTC degradation with HAO as catalyst under VLI.
