# Supplementary material for: Novel Visible Light-Driven Ho2InSbO7/Ag3PO4 Photocatalyst for Efficient Oxytetracycline Contaminant Degradation
Source: Molecules. 2025 Aug 6;30(15):3289. doi: 10.3390/molecules30153289 (PMC12348672; doi:10.3390/molecules30153289)
Supplement: Supplementary file 1 [file molecules-30-03289-s001.zip › molecules-3784322-supplementary.pdf]

Novel Visible Light-Driven Ho_2InSbO_7/Ag_3PO_4 Photocatalyst for Efficient Oxytetracycline Contaminant Degradation

Jingfei Luan [1,2,*] and Tiannan Zhao [1]

1 School of Physics, Changchun Normal University, Changchun 130032, China; 15734278033@139.com (T.Z.).
2 State Key Laboratory of Pollution Control and Resource Reuse, School of the Environment, Nanjing University, Nanjing 210093, China
* Correspondence: jfluan@nju.edu.cn; Tel.: +86-199-5193-9498

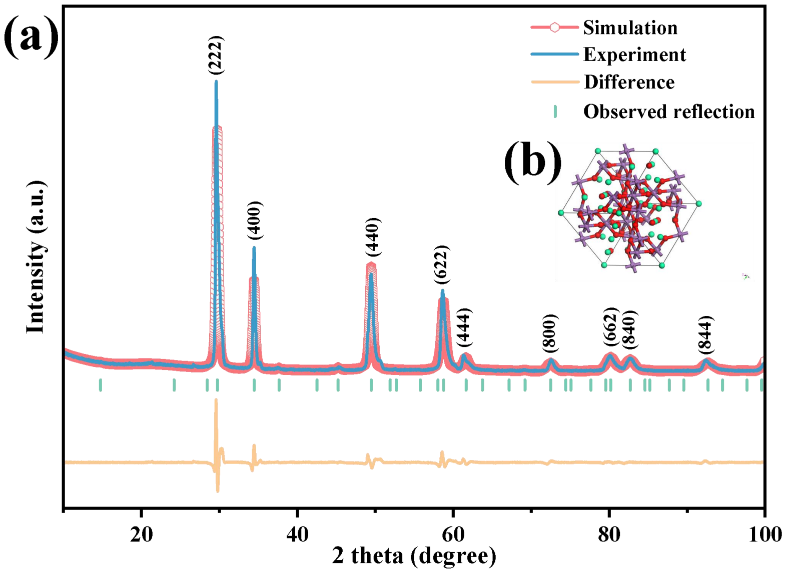

Figure S1. (a) XRD pattern and Rietveld refinement and (b) the atomic architecture (red atom: O; green atom: Ho; purple atom: In or Sb) of Ho_2InSbO_7.

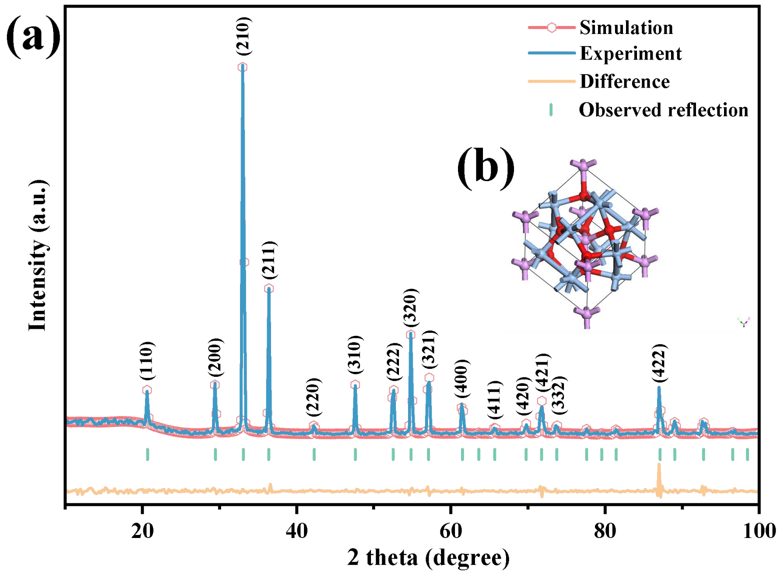

Figure S2. (a) XRD pattern and Rietveld refinement and (b) the atomic architecture (red atom: O; blue atom: P; pink atom: Ag) of Ag_3PO_4.

Table S1. Configurable properties of Ho_2InSbO_7 fabricated using solvothermal method.

**Atom**	**x**	**y**	**z**	**Occupation Factor**
Ho	0	0	0	1
In	0.5	0.5	0.5	0.5
Sb	0.5	0.5	0.5	0.5
O(1)	−0.175	0.125	0.125	1
O(2)	0.125	0.125	0.125	1

Table S2. Configurable properties of Ag_3PO_4 fabricated using solvothermal method.

**Atom**	**x**	**y**	**z**	**Occupation Factor**
Ag	0.25	0	0.50	1
P	0	0	0	1
O	0.1509	0.1509	0.1509	1

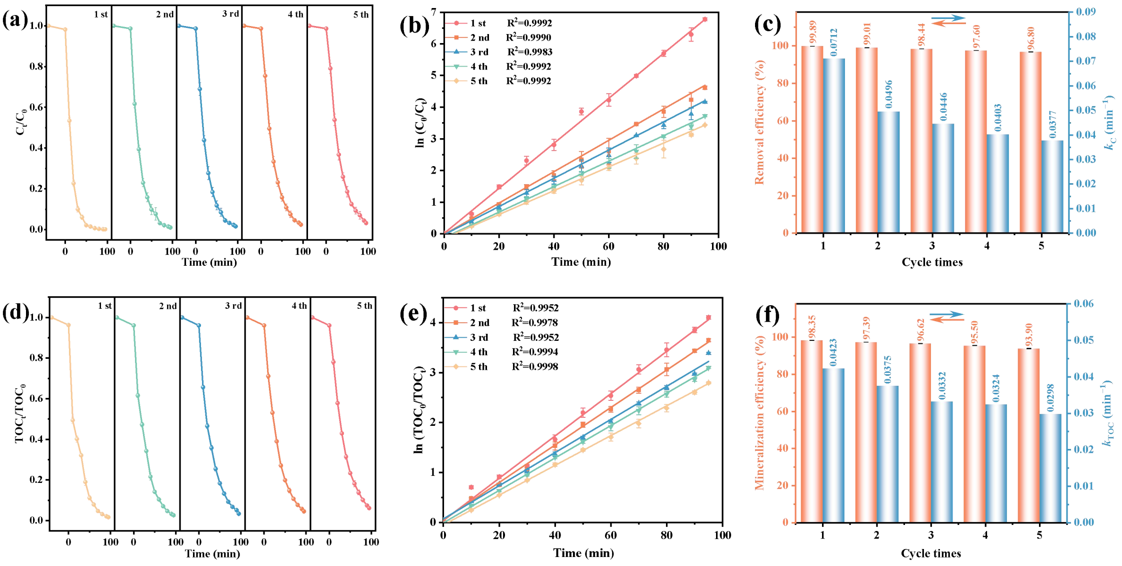

Figure S3. (a) Photodegradation; (b) kinetic curves, and (c) removal efficiencies and kinetic constants for five consecutive OTC degradation tests; (d) mineralization, (e) kinetic curves, and (f) mineralization efficiencies and kinetic constants for five consecutive TOC mineralization tests.

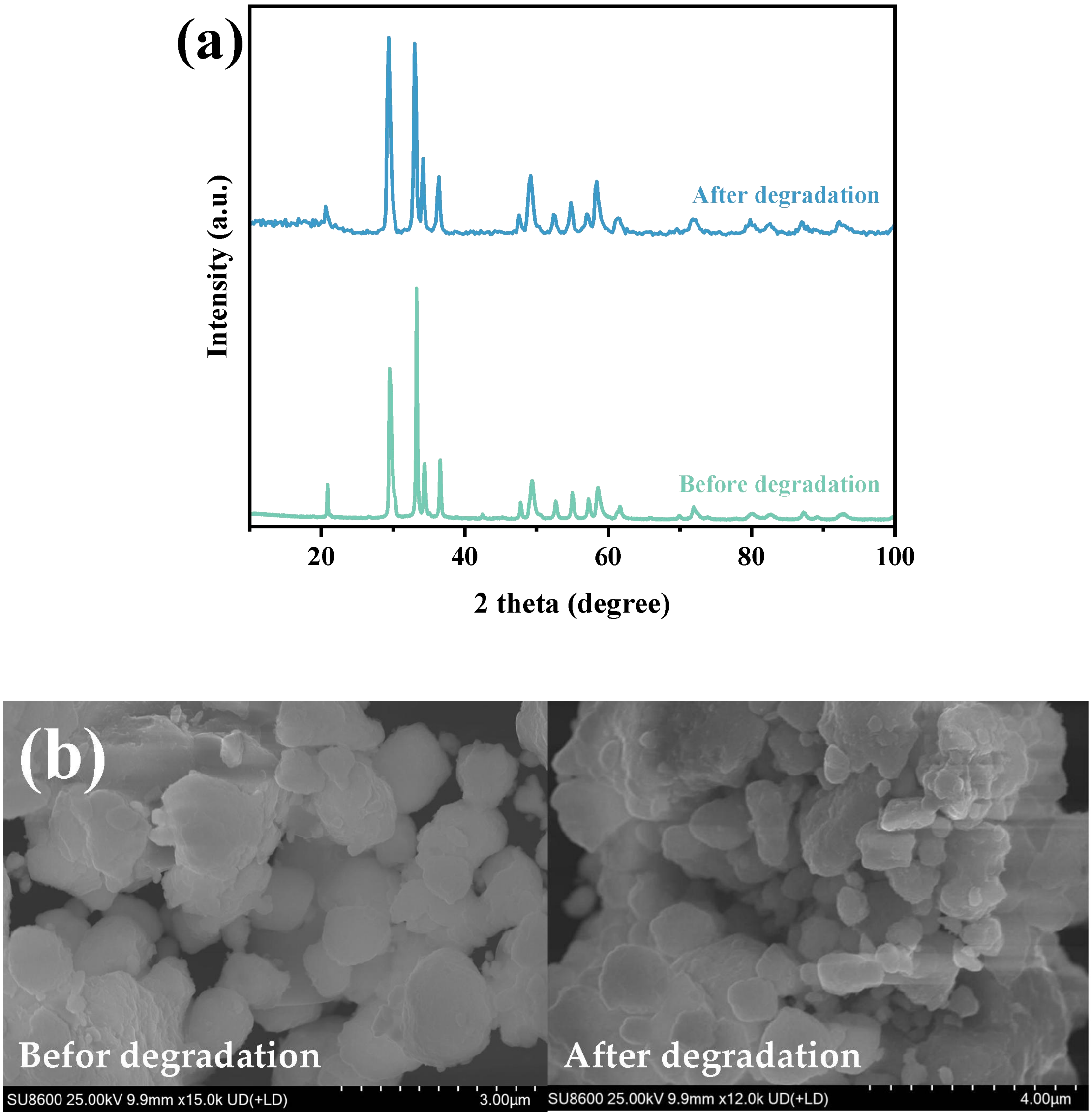

Figure S4. (a) XRD and (b) SEM patterns of the fresh and the used HAO

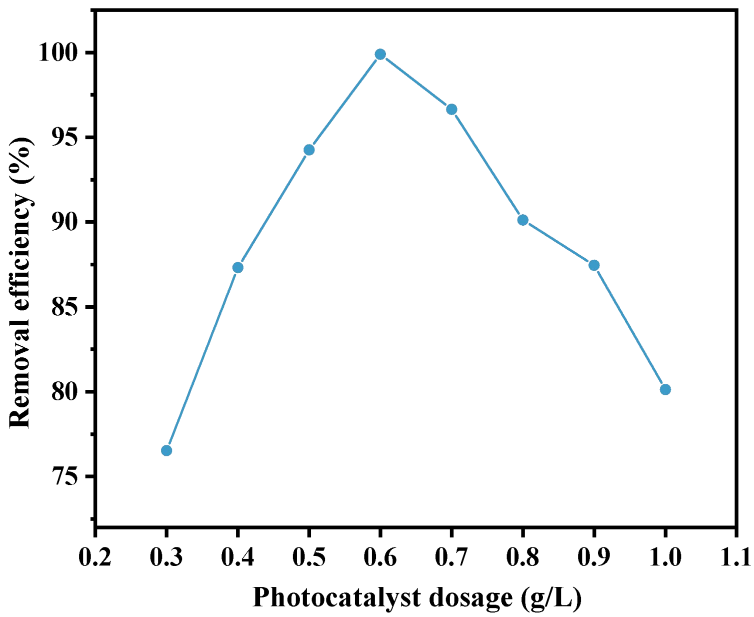

Figure S5. Impact of HAO dosage on removal efficiency of OTC.

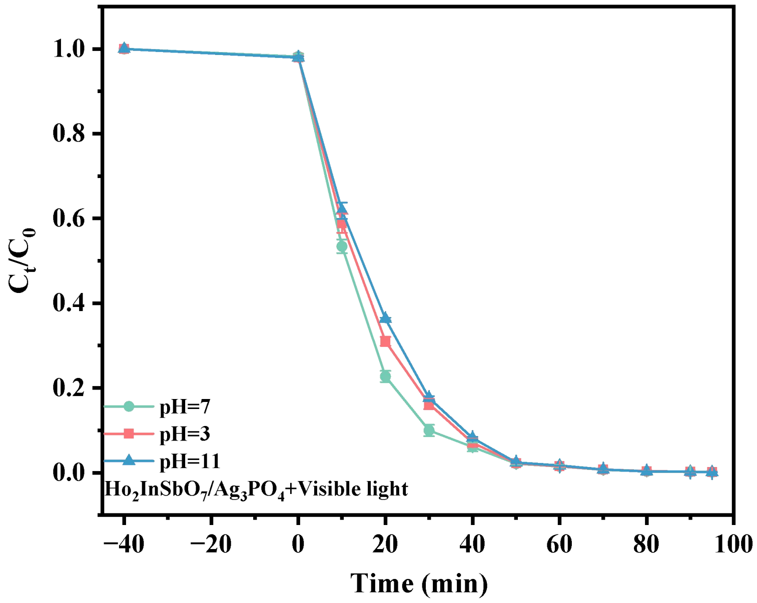

Figure S6. The effect of different pH values on OTC degradation with HAO as catalyst under VLI.

Section S1. Synthesis of N-Doped TiO_2

A total of 10 mL of tetrabutyl titanate was combined with 75 mL of absolute ethanol to create Solution A. Under magnetic stirring, Solution A was added dropwise to Solution B, which comprised a mixture of 15 mL of glacial acetic acid and 6.25 mL of double-distilled water. After stirring the combined Solutions A and B vigorously for 30 min, a 1 mol/L ammonia solution was introduced, maintaining a molar ratio of nitrogen to titanium (N/Ti) at 8%. The stirring was continued for an additional 120 min to ensure thorough mixing. The resultant mixture was allowed to equilibrate at room temperature for 24 h. Following this, the mixture was transferred to a drying oven and subjected to heating at 105 °C for 4 h. The solid obtained was then ground, and the resulting powder was calcined in a high-temperature furnace at 400 °C for 2 h, after which it was ground once more to yield nitrogen-doped titanium dioxide powder.

Section S2. Characterization

Crystallographic data were acquired through X-ray diffraction (XRD) utilizing a Shimadzu XRD-6000 diffractometer, based in Kyoto, Japan. Functional groups and chemical bonding were analyzed using Fourier-transform infrared spectroscopy (FTIR) with a WQF-530A spectrometer from Beifen-Ruili Analytical Instrument (Group) Co., Ltd. in Beijing, China. The interactions of chemical bonds were further examined through Raman spectroscopy using an INVIA0919-06 system provided by RENSHAW plx, based in Wotton-under-Edge, Gloucestershire, UK. The microstructural and morphological characteristics were investigated via transmission electron microscopy (TEM) with a Talos F200X G2 instrument from Thermo Fisher Scientific, located in Waltham, MA, USA. Surface topography was analyzed through scanning electron microscopy (SEM), utilizing a SU8010 model from Hitachi, based in Kyoto, Japan. Elemental analysis was carried out employing energy-dispersive spectroscopy (EDS). X-ray photoelectron spectroscopy (XPS) was conducted using a PHI 5000 VersaProbe instrument from UlVAC-PHI in Maoqi City, Japan, to analyze the surface chemical composition and oxidation states. Optical properties of the samples were assessed using ultraviolet-visible diffuse reflectance spectrophotometry (UV-Vis DRS) with a UV-3600 spectrophotometer also from Shimadzu Corporation. Additionally, the properties of photoelectrochemical were characterized with an FLS980 spectrophotometer from Edinburgh Instruments Ltd. in Edinburgh, UK. The specific surface area of the samples were determined with BSD-PM2 specific surface area and porosity analyzer from BEISHIDE Instruments Technology (Beijing) Co., Ltd. in Beijing, China. Lastly, electron paramagnetic resonance (EPR) spectroscopy was employed to detect free radicals in the samples, utilizing an A300 instrument from Bruker Corporation in Karlsruhe, Germany. Ultraviolet photoelectron spectroscopy (UPS) was performed to measure the ionization potential of the valence band with an Escalab 250 xi instrument from Thermo Fisher Scientific in Waltham, MA, USA.
